# Glucosamine prevents polarization of cytotoxic granules in NK-92 cells by disturbing FOXO1/ERK/paxillin phosphorylation

**DOI:** 10.1371/journal.pone.0200757

**Published:** 2018-07-17

**Authors:** Janja Božič, Veronika Stoka, Iztok Dolenc

**Affiliations:** 1 Department of Biochemistry and Molecular and Structural Biology, Jozef Stefan Institute, Ljubljana, Slovenia; 2 International Postgraduate School Jozef Stefan, Ljubljana, Slovenia; University of Leeds, Faculty of Medicine and Health, UNITED KINGDOM

## Abstract

Glucosamine (GlcN) is a naturally occurring derivative of glucose and an over-the-counter food additive. However, the mechanism underlying GlcN action on cells is unknown. In this study, we investigated the effect of GlcN on natural killer (NK) cells. We demonstrate that GlcN affects NK-92 cell cytotoxicity by altering the distribution of cathepsin C, a cysteine protease required for granzyme processing in cytotoxic granules. The relocation of cathepsin C due to GlcN was shown to be accompanied by a decrease in the intracellular enzyme activity and its extracellular secretion. Similarly, the relocation of endosomal aspartic cathepsin E was observed. Furthermore, we elucidated that repositioning of cathepsin C is a consequence of altered signaling pathways of cytotoxic granule movement. The inhibition of phosphorylation upstream and downstream of ERK by GlcN disturbed the polarized release of cytotoxic vesicles. Considerable changes in the ERK phosphorylation dynamics, but not in those of p38 kinase or JNK, were observed in the IL2-activated NK-92 cells. We found decreased phosphorylation of the transcription factor FOXO1 and simultaneous prolonged phosphorylation of ERK as well as its nuclear translocation. Additionally, a protein downstream of the ERK phosphorylation cascade, paxillin, was less phosphorylated, resulting in a diffuse distribution of cytotoxic granules. Taken together, our results suggest that dietary GlcN affects signaling pathway activation of NK-92 immune cells.

## Introduction

Glucosamine (GlcN; 2-amino-2-deoxy-d-glucose) is a dietary supplement often used by patients with osteoarthritis. However, clinical studies to date have not provided any evidence of its effectiveness in the treatment of hip and/or knee osteoarthritis [1]. GlcN does not affect fasting blood glucose levels, glucose metabolism, or insulin sensitivity at any oral dose level in healthy individuals [2], while its intestinal absorption allows it to reach high cellular concentrations [3]. Comparison of orally and intravenously administered GlcN showed that its oral ingestion leads to only four times lower bioavailability of this compound because a considerable fraction of GlcN undergoes first-pass metabolism in the liver [3]. GlcN enters cells through glucose transporter GLUT2, which has a much higher affinity for GlcN than for glucose [4].

Previous studies on aging animal models demonstrated that GlcN extends the lifespan of the evolutionary distinct species by mimicking a low-carbohydrate diet [5] or inhibiting tumor growth, when used intravenously [6]. Increased accumulation of GlcN in cells leads to inhibition of protein biosynthesis and irreversible damage to organelles in the tumor, but not in healthy cells [7]. GlcN showed neuroprotective and anti-inflammatory effects in a model of middle cerebral occlusion [8]. There is evidence that GlcN can regulate the production of nitric oxide (NO) in LPS-stimulated macrophages by regulating expression of inducible NO synthase [9]. Furthermore, GlcN can regulate expression of other genes, for instance, it suppresses the expression of proinflammatory cytokine genes by modification of *O*-GlcNAc in synovial cell line [10] and inhibits interleukin-1β-induced nuclear factor-κB activation in chondrocytes [11]. GlcN exhibited a therapeutic effect in an animal model of multiple sclerosis [12]. Additionally, it suppresses activation of T-lymphoblast and dendritic cells *in vitro* [7], and inhibits the cytotoxic effect of natural killer (NK) cells, which show cytotoxic activity against cancer and virus-infected cells [13], in a dose-dependent manner [14].

NK cells are activated by a number of cytokines or activating receptors [15], triggering highly coordinated activities that result in polarization of granules, followed by secretion of their contents into the immunological synapse [16]. This process was shown to be activated by the SRC family kinases, which induce the activation of two signaling pathways: ERK and JNK [17], and at least one of them is required for polarization of the microtubule-organizing center (MTOC) [17, 18] controlled by paxillin [19]. This center enables migration of cytolytic granules to the immunological synapse, located between the NK cell and the target cell. These granules release perforin and granzymes into the synaptic cleft, leading to apoptosis of the target cell [17].

Granules are secretory vesicles containing perforin, cathepsin C, and granzymes in addition to other molecules [20]. Perforin oligomerizes to form pores in the plasma membrane of the target cell [21], cathepsin C [22] is a tetrameric cysteine protease [23] that activates granzymes by removing dipeptides from their N-termini [24], and granzymes are serine proteases that induce apoptosis in target cells [25]. In addition to cathepsin C, cytotoxic granules contain other cysteine cathepsins [26, 27] such as cathepsin L, W, H, and the aspartic cathepsin D [28–31]. Cathepsin E is an endosomal aspartic protease of the pepsin superfamily with different functions and is highly homologous to the lysosomal aspartic protease cathepsin D [30].

We hypothesized that the immunosuppression that develops following administration of GlcN is a result of alterations in the signaling pathways regulating cellular vesicle transport. Therefore, in this study, we investigated the effects of GlcN on the cytotoxic activity of NK-92 cells and granule polarization.

## Materials and methods

### Ethics statement

The animal facilities in our department at J. Stefan Institute were approved by the decree UVHVVR, OU Ljubljana No. U34401-24/2013/9, date 30.10.2013, allowing to race laboratory mice. Procedures for animal care and experiments were conducted in conformity with the “Guide for the Care and Use of Laboratory Animals”. The Ethics committee for experiments with animals at the Administration of the Republic of Slovenia for food safety, veterinary and plant protection approved the protocol (Approval No. U34401-12/2014/4).

### Antibodies and reagents

Mouse monoclonal antibodies against human perforin (pf-344) were purchased from Mabtech (cat. no. 3465-6-250, Stockholm, Sweden), while the monoclonal rabbit anti-ERK (137F5, cat. no. 4695), anti-FOXO1 (C29H4, cat. no. 2880), and anti-paxillin (D9G12, cat. no. 12065), polyclonal rabbit anti-p38 (cat. no. 9212) and anti-JNK (81E11, cat. no. 9252) together with monoclonal rabbit anti-phospho-ERK (Thr202/Tyr204; D13.14.4E, cat. no. 4370), anti-phospho-JNK (Thr183/Tyr185; cat. no. 4668) and anti-phospho-p38 (Thr180/Tyr182; cat. no. 9211), and mouse monoclonal anti-phospho-Thr (42H4, cat. no. 9386), and anti-*O*-GlcNAc (CTD110.6, cat. no. 9875) antibodies were all purchased from Cell Signaling Technology (Danvers, MA, USA). Mouse monoclonal anti-β-actin antibody (AC-15, A1978) was obtained from Sigma-Aldrich (Darmstadt, Germany), anti-mouse (cat. no. 115-035-068) and anti-rabbit (cat. no. 111-035-045) horseradish peroxidase (HRP)-conjugated secondary antibodies were from Jackson Immunoresearch (West Grove, PA, USA), Alexa Fluor 488 anti-mouse (cat. no. A11029) and anti-rabbit (cat. no. A11034), and Alexa Fluor 546 anti-mouse (cat. no. A21045) antibodies were purchased from Invitrogen (Carlsbad, CA, USA). Goat anti-cathepsin E antibodies (cat. no. AF1294) were purchased from R&D Systems (Minneapolis, MN, USA), while mouse anti-cathepsin C antibodies (D-6, cat. no. sc74590) and anti-phospho-Ser antibodies (16B4, cat. no. sc-81514) were from Santa Cruz Biotechnology (Santa Cruz, CA, USA). d-(+)-GlcN hydrochloride and *N*-acetyl glucosamine (GlcNAc) were purchased from Sigma-Aldrich.

### Recombinant cathepsin activity assay

The activity of recombinant cathepsins C, L, and E was determined by analyzing the hydrolysis of the fluorogenic substrates H-Gly-Phe-AMC and Z-Phe-Arg-AMC (Bachem, Bubendorf, Switzerland), and KYS-1 (Peptide Institute Inc., Osaka, Japan), respectively. Recombinant cathepsin C [32, 33] was assayed at pH 6.0, as previously described [34]. The activity of recombinant cathepsin L [35] was measured at pH 5.5, as described [36], while that of recombinant cathepsin E [37] was determined at pH 5.0 [37].

### Cell cultures and animals

Human NK cell line derived from blood, mononuclear cells, NK-92, kindly provided by E. Vivier (Marseille, France), was cultured in RPMI-1640 (Sigma-Aldrich) with 20% heat-inactivated fetal bovine serum (hiFBS) supplemented with 100 U/mL of recombinant IL2 (Cell Sciences). Mouse primary NK cells were obtained from spleen of FVB/NJ mouse strain (JAX stock #001800) as previously described [38], negatively selected by magnetic selection using a MACS column (Miltenyi Biotec, Auburn, CA, USA) and cultured in RPMI-1640 with 20% heat-inactivated fetal bovine serum (hiFBS) supplemented with 100 U/mL of recombinant IL2 (Cell Sciences). Human hematopoietic malignant cell line K562, kindly provided by E. Vivier (Marseille, France) was cultured in RPMI-1640 supplemented with 10% hiFBS. Mouse mammary gland tumor cell line 4T1 (ATCC) was cultured in DMEM (Sigma-Aldrich) supplemented with 10% FBS. All cell cultures were maintained at 37°C and 5% CO_2_.

### Cytotoxicity assay

NK cell cytotoxicity assay was performed using fluorescence-activated cell sorter (FACS) as described previously [39], with some modifications. The number of effector NK-92 cells was determined 1 day before testing, and the cells were incubated overnight with GlcN at concentrations ranging from 0.1 mM to 10 mM. The following day, the target K562 cell numbers were determined, and to distinguish them from the effector cells, they were labeled with 30 μM of 3,3′-dioctadecyloxacarbocyanine (DiOC18) (Sigma-Aldrich), a green fluorescent membrane stain. Afterward, the effector and labeled target cells were mixed at the desired ratio, 10:1, and these cells, together with the effector cells alone, were incubated for 4 h at 37°C and 5% CO_2_. Following incubation, the cells were centrifuged and the pellets resuspended in 0.5 μM propidium iodide (Sigma-Aldrich) in phosphate-buffered saline (PBS) to evaluate cell viability. The analysis was performed using a FASCalibur flow cytometer (Becton-Dickinson, Franklin Lakes, NJ, USA), while the data acquisition was performed using Cellquest software. Two-parameter cytograms were plotted to discriminate between different cell types and live/dead cells. To calculate the cytotoxicity, the percentage of dead target cells in presence of the effector cells was corrected for the spontaneous target-cell death in absence of the effector cells, as follows:

$$cytotoxicity\;(\%)=\frac{(dead\;target\;(\%)-spontaneus\;death\;(\%))\times 100}{\left(100-spontaneus\;death\;(\%)\right)}$$

### Cellular cathepsin activity assay

NK-92 cells were incubated overnight with 10 mM GlcN, and then incubated for 4 h in serum-free medium containing 0.5% bovine serum albumin (BSA) and activated for 30 min with 1000 U/mL of IL2. The cells were then lysed in a buffer containing 50 mM phosphate buffer pH 6.0, 150 mM NaCl, 1 mM EDTA. 0.5% NP-40, 0.1% sodium dodecyl sulfate (SDS), and 2.5 mM DTT. The conditioned medium samples were collected as well, 10-times concentrated, and normalized to obtain equal volumes. The protein contents of the whole-cell lysates were normalized by the Bradford assay. The activity was measured in 100 mM sodium acetate buffer (pH 5.5) with 1 mM EDTA and 5 mM DTT. We used three different substrates: H-Gly-Phe-AMC, specific for cathepsin C; Z-Phe-Arg-AMC, specific for cysteine cathepsins; and KYS-1, specific for cathepsin E, in our analyses.

### Cell activation, immunoprecipitation, and immunoblotting

Control NK-92 cells or NK-92 cells treated with 10 mM GlcN overnight at 37°C were incubated in serum-free and IL2-free media for 4 h at 37°C to reduce background phosphorylation. To initiate NK-92 activation, high concentrations of IL2 were added (1000 U/mL) and the cells incubated for the indicated periods up to 30 min at 37°C. For whole-cell lysates, the cells were pelleted at 250 ×*g* at 4°C and lysed in lysis buffer containing 20 mM Tris-HCl (pH 7.4), 150 mM NaCl, 1 mM EDTA, 1% NP-40, 10% glycerol, 1 mM Na_3_VO_4_, 10 mM NaF, 10 mM β-glycerolphosphate, protease inhibitor cocktail (Sigma-Aldrich), and phosphatase arrest cocktail (G-Biosciences, St. Louis, MO, USA). Equal amounts of total proteins were resolved by 12.5% SDS-polyacrylamide gel electrophoresis (PAGE) and electrotransferred to polyvinylidene difluoride membranes. For immunoprecipitation, antibodies against FOXO1 and paxillin were added to the cell lysates and incubated overnight. Afterward, Protein G Sepharose 4 Fast Flow (GE Healthcare) was added and the samples incubated for 3 h. The beads were then washed five times, resuspended in 3× SDS loading buffer, and heated for 5 min to 100°C. For determination of the *N*-glycosylation type, the whole-cell lysates were deglycosylated by PNGase F and Endo H (New England Biolabs, UK) according to the manufacturer’s instructions. Nuclear and cytoplasm fractions were prepared by REAP method as previously described [40]. The blots were probed with anti-cathepsin E, anti-cathepsin C, anti-perforin, anti-phosphorylated kinases, anti-ERK, -JNK, -p38, anti-phospho-Ser, anti-phospho-Thr antibodies, and anti *O*-GlcNAc antibodies.

### Fluorescence confocal microscopy and image analysis

NK-92 cells and K562 cells, with or without 10 mM GlcN, were incubated together for 60 min at 37°C, and cytospun on glass slides. The cells were fixed in 4% paraformaldehyde, permeabilized (0.1% Triton X-100), and blocked with 3% BSA in PBS. The cells were then labeled with primary antibodies, washed with PBS, and labeled with secondary Alexa Fluor antibodies. The slides were washed again with PBS and mounted with ProLong Gold antifade with DAPI (Invitrogen). The same procedure was performed on primary mouse NK cells and target cancer 4T1 cells, with modification. 4T1 cells were seeded on a coverslip, and the next day, primary NK cells were added in serum-free medium, which allows them to attach to the coverslip. Imaging was performed using an inverted confocal laser scanning microscope, LEICA TCS SP5 X, with an oil objective HCX PL APO 60× (N.A. = 1.4).

Colocalization micrographs of cathepsin C with cathepsin E, and cathepsin C with perforin were evaluated by Leica software LAS AF. The polarization of lytic granules was scored for at least 20 conjugates per slide in randomly selected fields, in three independent experiments. Conjugates were analyzed using ImageJ [41], by dividing the NK-92 cells into four equal quarters with Draw concentric quadrants macro (ImageJ). The integrated density of perforin signals in the quarter nearest to the target cell was analyzed, and cells with more than 50% of signal in the quarter nearest to the K562 cells were considered polarized.

Nuclear translocation rate of the phosphorylated ERK was determined using the IL2-activated cells. Cells were activated for 60 min, fixed, and then treated as described in previous sections. The analysis was performed as described previously [42], with some modifications for the analysis of nuclear translocation in single cells. Obtained intensities were sorted by the size of the nuclear or cytoplasmic region of interest, to obtained intensity per area, and the ratios of nuclear to cytoplasmic intensity per area were compared.

### Statistical analysis

Data were analyzed and plotted using Excel 2016 (Microsoft), ImageJ, and Corel Draw, and presented as mean values ± standard deviations (SD) of the results obtained in at least three replicates, unless indicated otherwise.

## Results

### GlcN, but not GlcNAc, decreases NK-92 cell cytotoxicity

**Fig 1:**
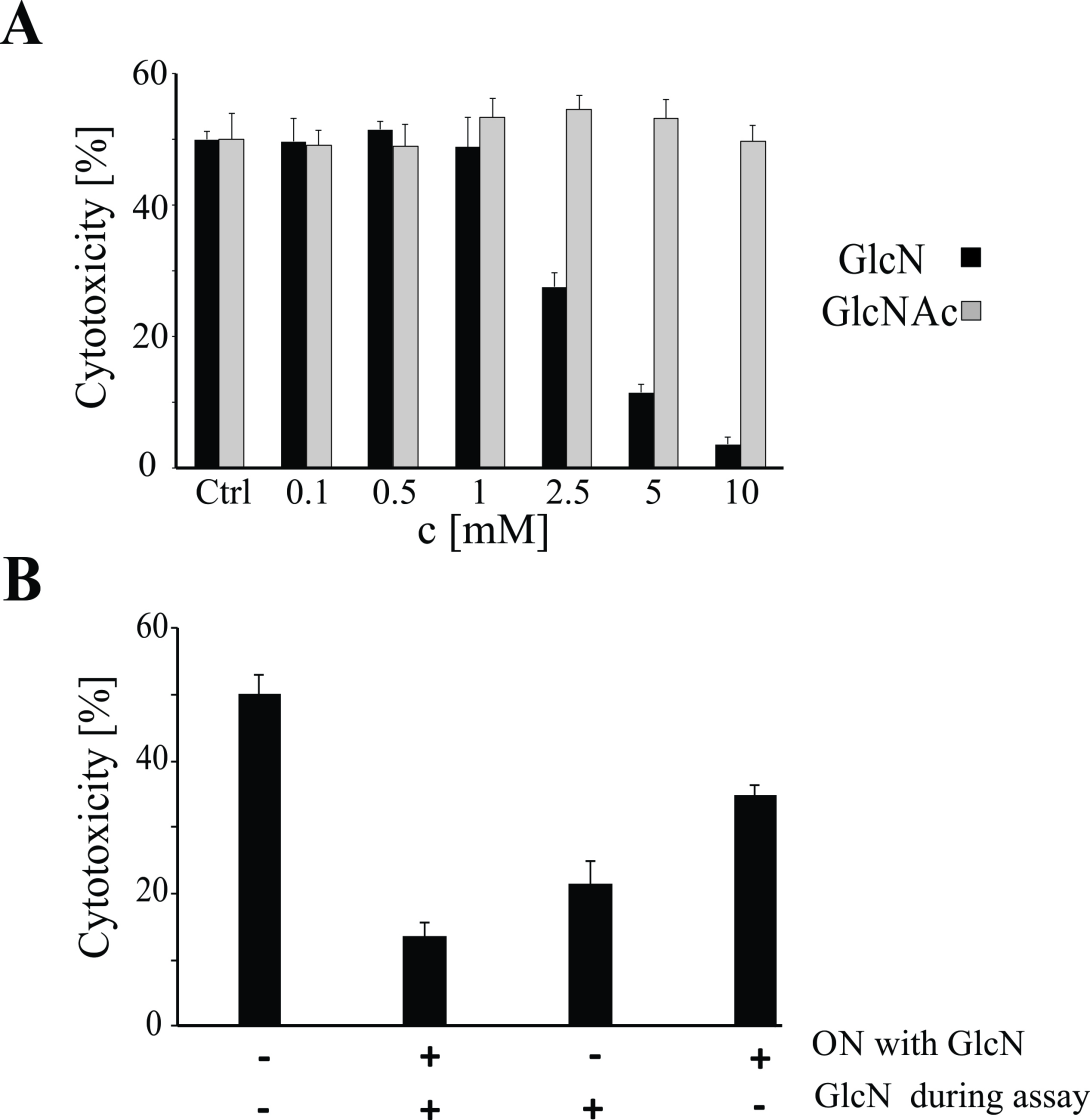
Glucosamine (GlcN) Suppresses the Cytotoxic Activity of NK-92 Cells Against K562 Cells. (A) Cytotoxicity of NK-92 cells against K562 cells, following treatment of the cultures overnight (ON) with different concentrations of GlcN or *N*-acetyl GlcN (GlcNAc). (B) Cytotoxicity of NK-92 cells against K562 cells after ON pretreatment with GlcN, followed by 5-mM GlcN supplementation during the assay. All experiments were performed independently five times. Error bars represent standard deviations.

We examined the effects of GlcN and GlcNAc concentrations ranging from 0.1 to 10 mM, on the NK-92 cell cytotoxicity against K562 cells (Fig 1A), and observed that GlcN treatment led to decrease in the NK cell cytotoxicity in a dose-dependent manner. Cytotoxicity was shown to decrease with GlcN concentrations above 2.5 mM, while complete inhibition was achieved at 10-mM concentration. GlcNAc did not affect the NK cell-mediated cytotoxicity towards K562 cells (Fig 1A). GlcN and GlcNAc did not affected the viability of neither K562 nor NK cells alone. The cytotoxicity of NK-92 cells against the target cells was restored after removing GlcN from the medium (Fig 1B). Additionally, addition of GlcN without an overnight pretreatment led to decrease in the calculated cytotoxicity, from 50% to 20%. In comparison, the overnight treatment alone resulted in a cytotoxicity of 35%. However, when the cells were pretreated overnight with GlcN and the amino-sugar was present during the assay, the cytotoxicity of the NK cells dropped to approximately 10% of that in the controls.

### GlcN does not affect cathepsin activity and *N*-glycosylation *in vitro*

**Fig 2:**
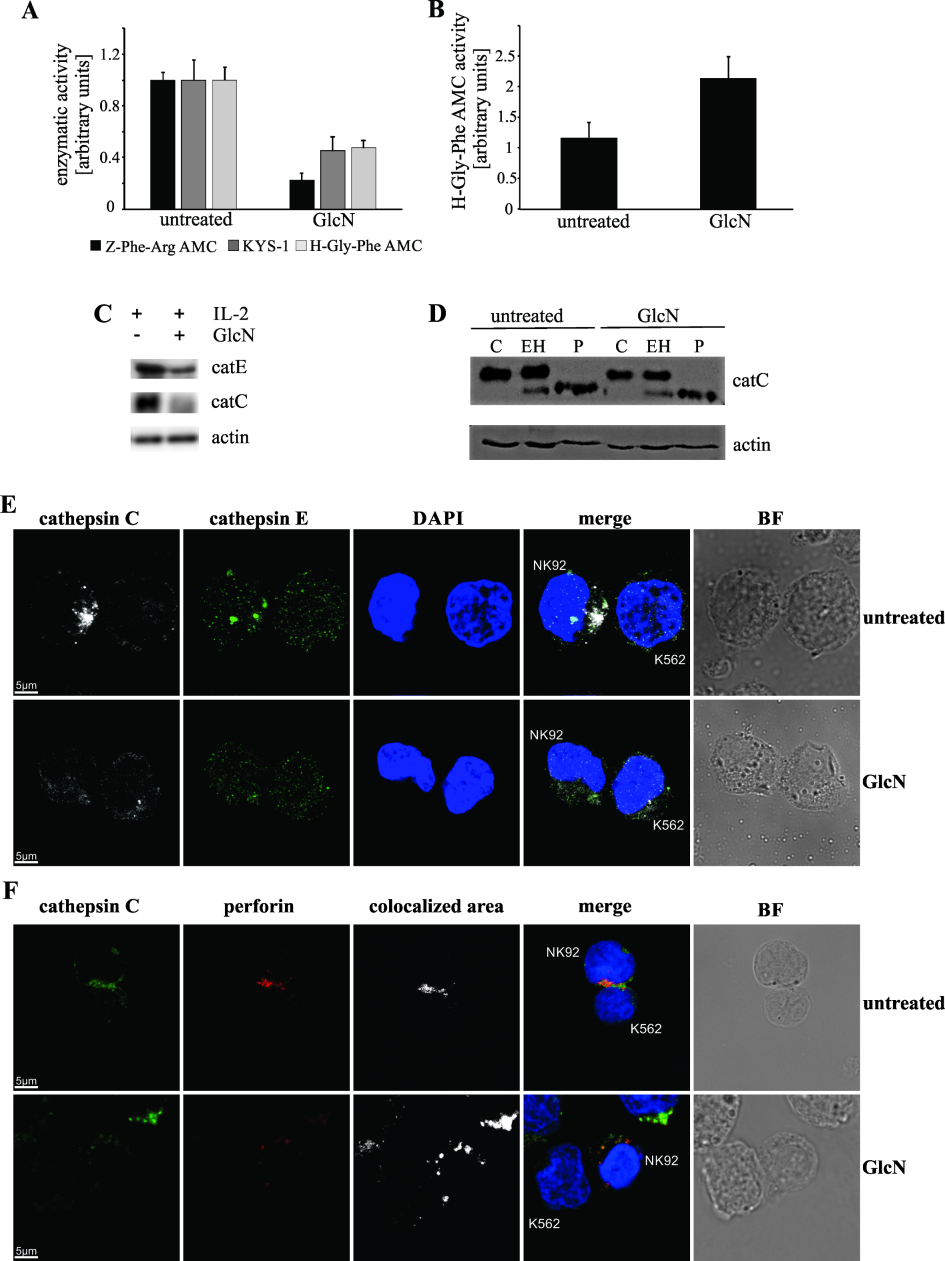
Glucosamine (GlcN) Treatment Affects Cathepsin C and E Intracellular Levels and Localization in NK-92 cells. (A) Activities of specific cysteine cathepsins (substrate, Z-Phe-Arg-AMC), cathepsin E (KYS-1), and cathepsin C (H-Gly-Phe-AMC) determined in NK-92 cells treated with or without GlcN. Enzyme activities are presented as fold changes relative to the activity of these enzymes in the untreated cells. (B) Secreted cathepsin C activity following GlcN treatment. Results are presented as fold changes relative to the values obtained for the controls samples. Error bars represent standard deviations of the results obtained from five independent experiments. (C) Cathepsin E and C levels in the lysate of the untreated and GlcN-treated NK-92 cells. β-actin levels were used for normalization. (D) Cathepsin C *N*-glycosylation type in the GlcN-treated and untreated cells determined using EndoH (EH) and PNGaseF (P). (E) Subcellular localization of cathepsins C (white) and E (green) in NK-92 cells cultured with K562 cells, untreated or treated with GlcN. Cell nuclei were stained with DAPI, the right panel represent bright- filter (BF). Merged images show the overlapping signals. (F) Cathepsin C (green) and perforin (red) colocalization in GlcN-treated and untreated cells. The colocalized area panel shows colocalized area calculated by the LAS AF software.

Initially, we determined the activity of cathepsins C, L, and E in cell lysates and conditioned medium, using the fluorogenic substrates H-Gly-Phe-AMC, Z-Phe-Arg-AMC, and KYS-1, respectively. However, following treatment with GlcN, the activities of these enzymes in NK-92 cells were shown to decrease in comparison with those in the control lysates (Fig 2A). The activity of intracellular cathepsin C and E decreased by more than 50% in presence of GlcN, compared with those in the controls. The experiments using Z-Phe-Arg-AMC, a general substrate of cysteine cathepsins [36], demonstrated that the total cathepsin activity decreased by approximately 80% compared with that in the control samples. Afterward, we monitored the activity of the secreted enzymes and observed that the activity of the secreted cathepsin C, based on the hydrolysis of H-Gly-Phe-AMC, increased following GlcN treatment, in comparison with that in the controls (Fig 2B). No activity of cathepsin E or L was detected in the conditioned media. Western blot analyses confirmed that, following treatment with GlcN, intracellular levels of cathepsins C and E were reduced (Fig 2C). To exclude GlcN as an inhibitor of these enzymes, we measured the *in vitro* activity of purified recombinant cathepsins C, L, and E. We found that the substrate degradation rates remained unchanged, showing that GlcN does not affect the activity of the individual enzymes (data not shown).

Since all cathepsins are *N*-glycosylated, we examined whether GlcN treatment affects the type of *N*-glycosylation. A complex type of *N*-glycosylation of cathepsin C was observed in the NK-92 cells, and it did not change following GlcN treatment (Fig 2D).

### GlcN alters localization of cathepsins C and E

Localization of cathepsins C and E in the NK-92 and K562 cells was determined by confocal fluorescence microscopy (Fig 2E). A considerable difference in the cellular distribution of granules was observed. Cathepsin C is usually located in the lytic granules, which we confirmed with colocalization of cathepsin C with perforin (Fig 2F). In the untreated cells, lytic granules were focused toward the target cells. On the contrary, in presence of GlcN, these granules were distributed evenly in the cytoplasm, which was accompanied by changes in the localization of endosomes with cathepsin E, which were also not directed toward the target cells.

### GlcN prevents lytic granule polarization

**Fig 3:**
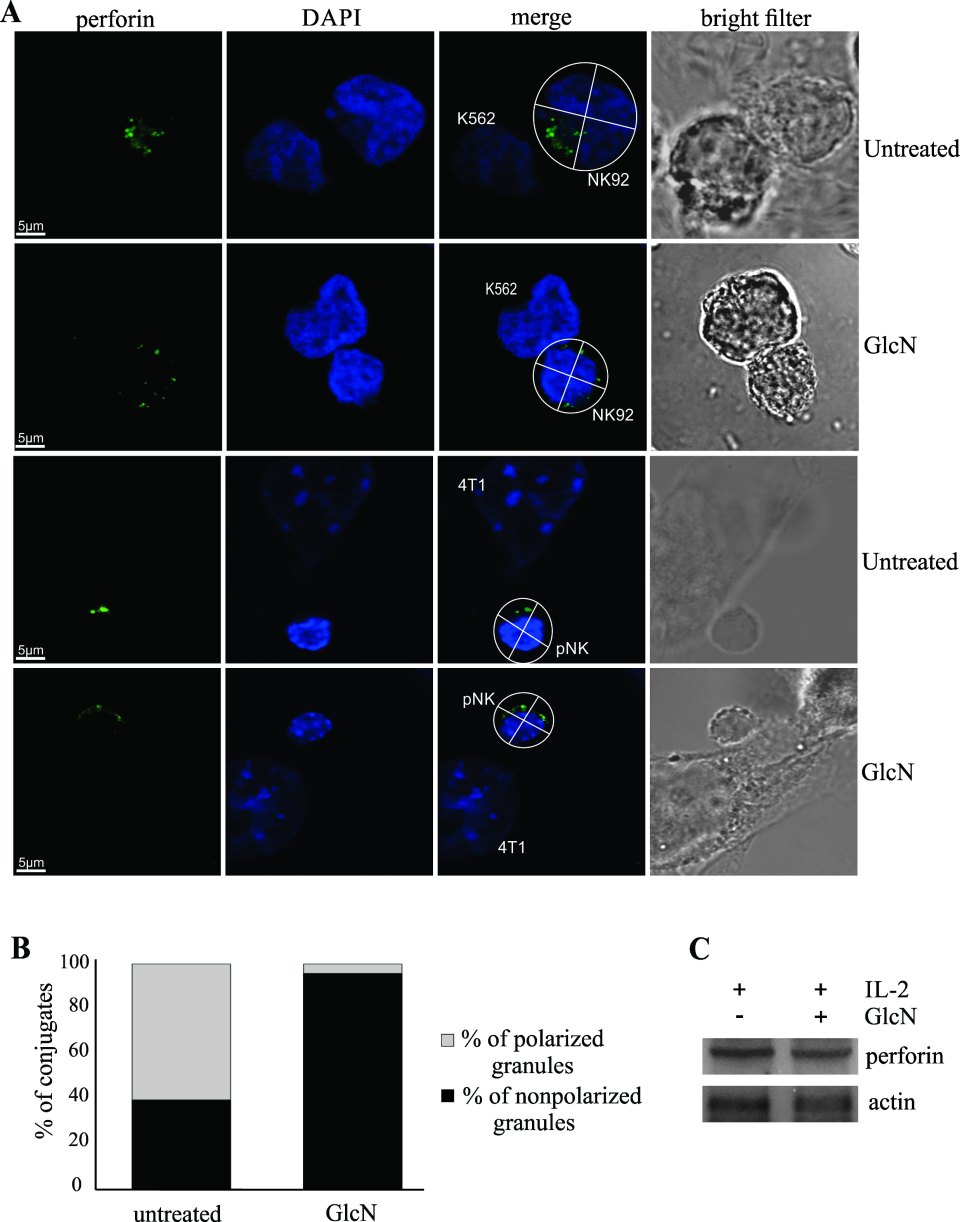
Glucosamine (GlcN) Prevents Lytic Granule Polarization in NK Cells. (A) Polarization of granules expressing perforin (green) in the NK-92 and K562 cell conjugates, in presence or absence of GlcN (upper panels). Representative images of at least 60 NK-92/K562 conjugates are shown. Polarization of granules expressing perforin (green) in the mouse primary NK cell and 4T1 cell conjugates, in presence or absence of GlcN (lower panels). Representative images of at least 20 NK/4T1 conjugates are presented. Conjugates were considered polarized when the perforin signal was located in the quarter of the NK cell nearest to the target cell (merged image). Right, bright-filter images, for detection of the conjugates. (B) Distribution analysis, showing the percentage of NK-92 and K562 conjugates with polarized lytic granules in the untreated and GlcN-treated cells. (C) Western blots of NK-92 cell lysates untreated and treated with GlcN, showing the amount of perforin.

We incubated the control and GlcN-treated NK-92 cells with K562 target cells, and fixed and stained them for perforin (Fig 3A). Mouse primary NK cells and mouse cancer 4T1 cell line were treated in the same manner (Fig 3A). Afterward, we analyzed the distribution of granules in the NK cells, since the membranes between the NK cell and target cell in contact appeared flat. Individual NK cells were divided into four quarters, and the number of granules in the quarters in contact with the target cell was analyzed [43]. In 60% of all control conjugates, but only 2% of the GlcN-treated conjugates, granules were observed to be polarized (Fig 3B). Despite the distributed granules in the GlcN-treated cells, the amount of perforin in these cells stayed unchanged (Fig 3C).

### GlcN alters FOXO1, ERK, and paxillin phosphorylation

**Fig 4:**
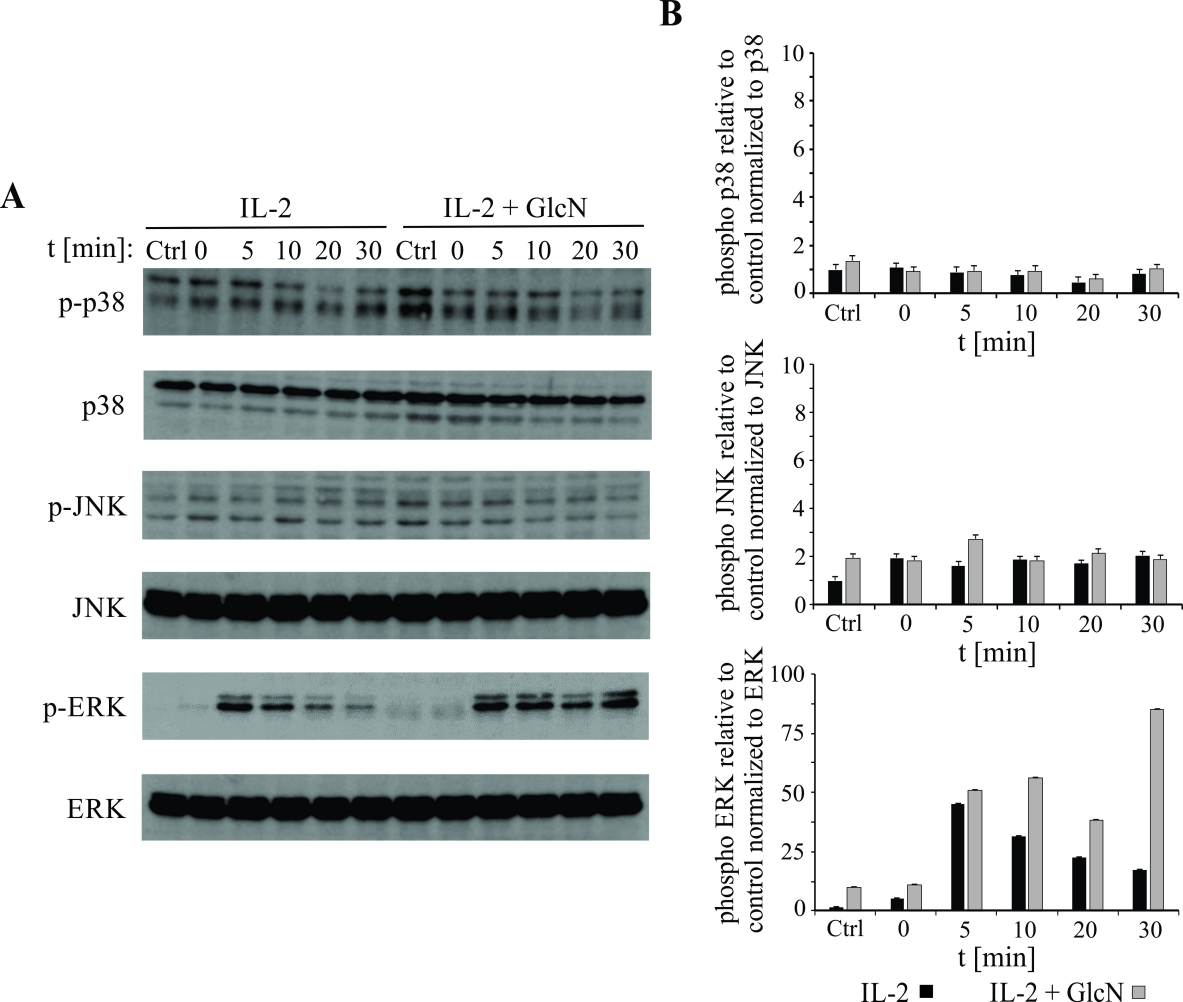
Glucosamine (GlcN) Prolongs ERK Phosphorylation in NK-92 cells. (A) Representative western blots showing the expression of phosphorylated P38, JNK, and ERK, and total MAPK protein levels in NK-92 cells treated with IL2 alone or in combination with GlcN. (B) Quantification of phosphorylated P38, JNK, and ERK levels in cells treated with IL2 alone or in combination with GlcN, normalized to the total protein levels. Error bars represent standard deviations.

The mitogen-activated protein kinase (MAPK) family is activated by stimulation of NK cells, and its members play important roles in the movements of intracellular granules [44–46]. We measured JNK, ERK, and P38 phosphorylation levels following IL2 stimulation of NK-92 cells in presence or absence of GlcN (Fig 4). IL2 treatment did not induce activation of either JNK or P38 kinase. However, it transiently activated ERK, with the phosphorylation levels peaking 5 min after initiation of the treatment, followed by rapid dephosphorylation. The addition of GlcN to the NK-92 cell culture induced prolonged phosphorylation of ERK, particularly ERK2.

**Fig 5:**
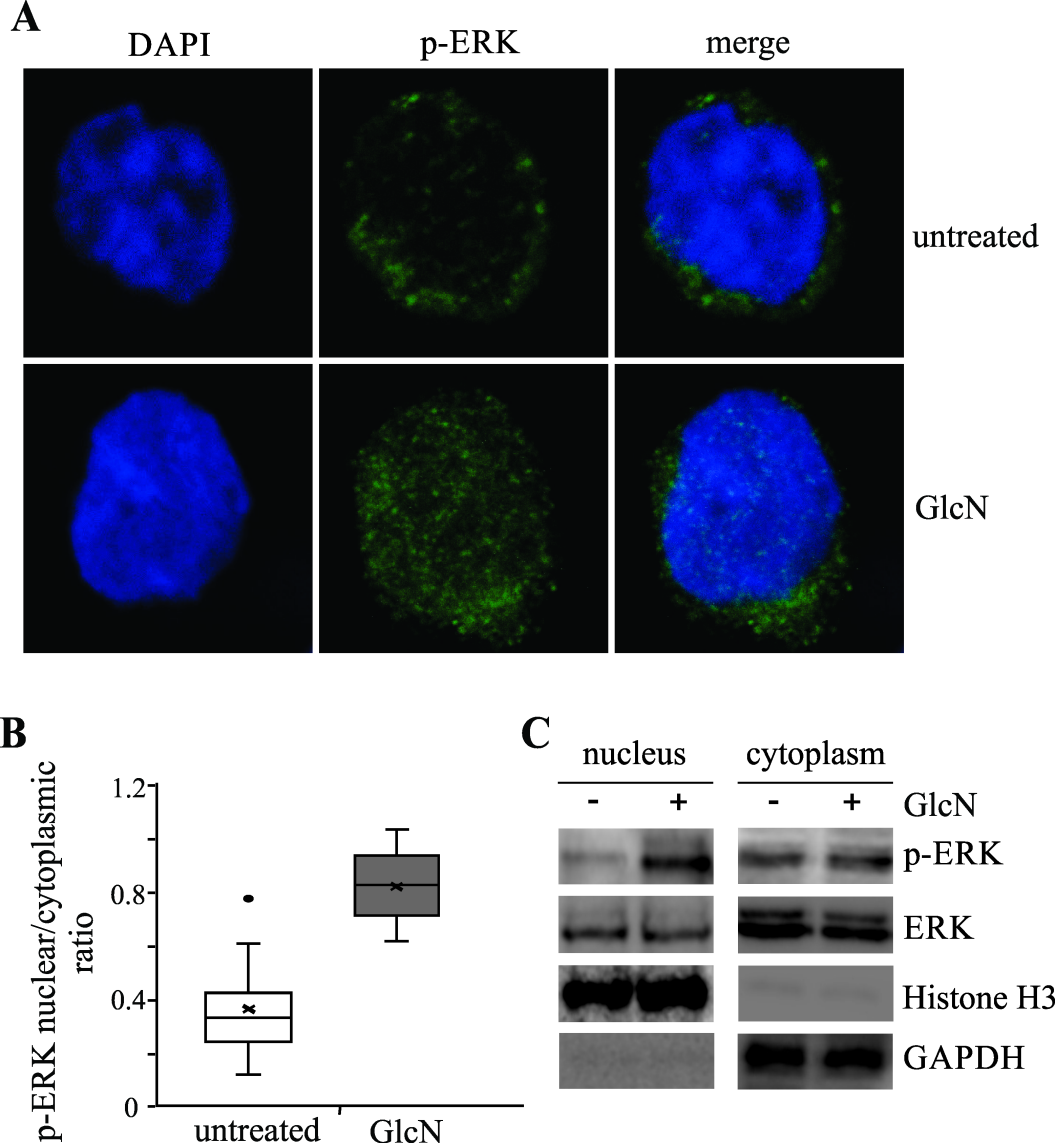
Glucosamine (GlcN) Triggers Nuclear Translocation of Phosphorylated ERK. (A) Representative images of phospho-ERK (green) expression in NK-92 cells treated with either GlcN and IL2 or IL2 alone. DAPI was used to stain the NK-92 cell nuclei. The merged image shows the overlapping DAPI and phospho-ERK signals. (B) Ratios of the nuclear and cytoplasmic concentrations of p-ERK in the GlcN-treated and untreated NK-92 cells. Sixty individual cells were analyzed in three independent microscopic slides. (C) Imunoblot shows the amount of p-ERK in the nuclei and cytoplasm of control and IL-2 activated (for 60 minutes) cells, treated with GlcN or left untreated. Histone H3 and GAPDH are showing that nuclear and cytosolic fractions were clear.

**Fig 6:**
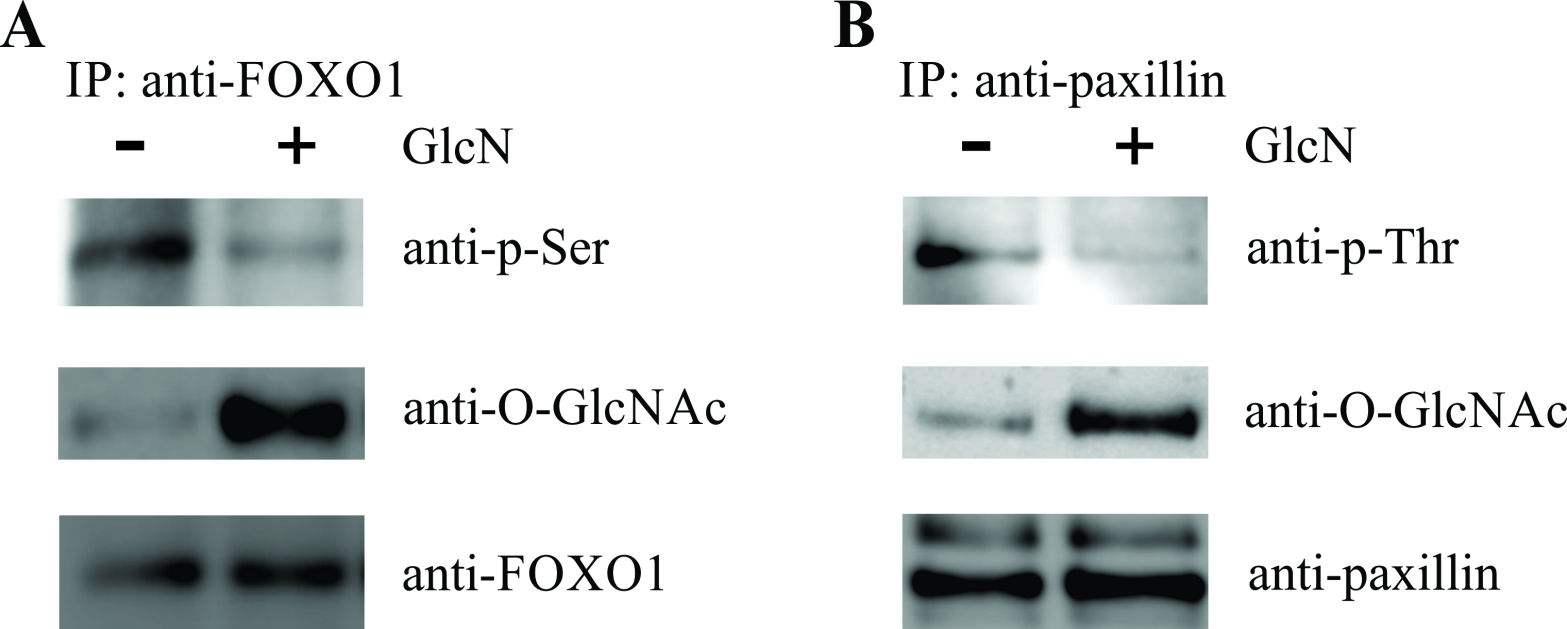
Glucosamine (GlcN) Decreases Phosphorylation of FOXO1 and Paxillin in NK-92 Cells. (A) Representative immunoblots show the immunoprecipitation of whole-cell lysates, using anti-FOXO1 antibodies, followed by immunoblotting with anti-phospho-Ser antibodies (upper), and reblotting with anti-*O*-GlcNAc (middle) antibodies, of the NK-92 cells untreated or treated with GlcN. (B) Whole-cell lysates were subjected to immunoprecipitation with anti-paxillin antibodies. Immunoprecipitates were analyzed by immunoblotting with anti-phospho-Thr antibodies (upper), and anti- *O*-GlcNAc (middle). Total paxillin levels were analyzed using the anti-paxillin antibodies (lower).

Prolonged phosphorylation was shown to lead to an increase in the nuclear translocation of phosphorylated ERK, as determined by confocal microscopy (Fig 5A). The differences in ratios of the nuclear and cytoplasmic concentrations between the untreated and GlcN-treated cells are presented in Fig 5B. We confirmed increased nuclear localization of p-ERK in GlcN-treated cells after 60 minutes of activation with IL-2, with western blotting of the nuclear and cytoplasmic fraction (Fig 5C). Following GlcN treatment of the NK-92 cells, we observed changes in the phosphorylation of an upstream transcription factor, FOXO1. Immunoprecipitation of FOXO1 showed a lower Ser phosphorylation level and increased *O*-glycosylation (Fig 6A), which may explain the prolonged phosphorylation of ERK. Therefore, we aimed to identify the main downstream moderator of the ERK pathway and observed that GlcN induces changes in the phosphorylation of paxillin, which is responsible for correct granule migration. Increased *O*-glycosylation of paxillin was accompanied by a decrease in the Thr phosphorylation levels following treatment with GlcN (Fig 6B).

## Discussion

In this study, we evaluated the inhibitory effects of GlcN on NK-92 cell cytotoxicity. Previously, a study demonstrated that GlcN has immunosuppressive effects on NK cells [14], whereas here, we showed that GlcN inhibits NK-92 cell cytotoxicity in a dose-dependent manner *in vitro*. In contrast, GlcNAc did not inhibit NK-92 cell cytotoxicity.

Previously, the localization and substrate specificity of cathepsins were confirmed to be involved in the regulation of NK cell cytotoxicity [28]. Cathepsin C, located in the NK cell granules [24], actively participates in the cellular cytotoxic activities by activating granzymes [47]. Similarly, the perforin-processing lysosomal protease cathepsin L [48] is present in lytic granules [48]. Although cathepsin E was previously detected in NK cells [49], its function and localization remain unclear.

Cathepsins C and E were shown not to colocalize in NK-92 cells, but addition of GlcN to the NK-92 cell medium affected the localization of both cathepsins. A decrease in cathepsin C protein levels in cell lysates (Fig 2C) and increase in its activity in the conditioned medium samples following treatment with GlcN (Fig 2D) indicate an increased secretion of this enzyme. Since cathepsin E content in the cell lysates decreased (Fig 2C), we assumed that GlcN induces its secretion as well, but were not able to detect cathepsin E activity in the conditioned media. This may be due to the low sensitivity of its substrate, KYS-1. Similarly, no activity of the secreted cathepsin L, using Z-Phe-Arg-AMC as a substrate, was detected, which may be explained by the enzyme instability in the neutral media [50]. To confirm that GlcN does not interfere with cathepsin C, L, and E activity, we analyzed the effects of GlcN on the activity of the purified recombinant cathepsins, and observed that it does not affect their activity *in vitro*.

GlcN was reported to interfere with *N*-linked glycosylation [51], while all cathepsins are *N*-glycosylated, and the type of *N*-glycosylation determines their intracellular trafficking [52, 53]. However, no changes in the type of *N*-glycosylation of cathepsin C in NK-92 cells were observed in presence of GlcN. We determined that polarization of endosomal cathepsin E is interrupted by treatment with GlcN, which may contribute to our understanding of the regulatory role of polarized endosomes in the development and activity maintenance in NK-92 cells [54].

**Fig 7:**
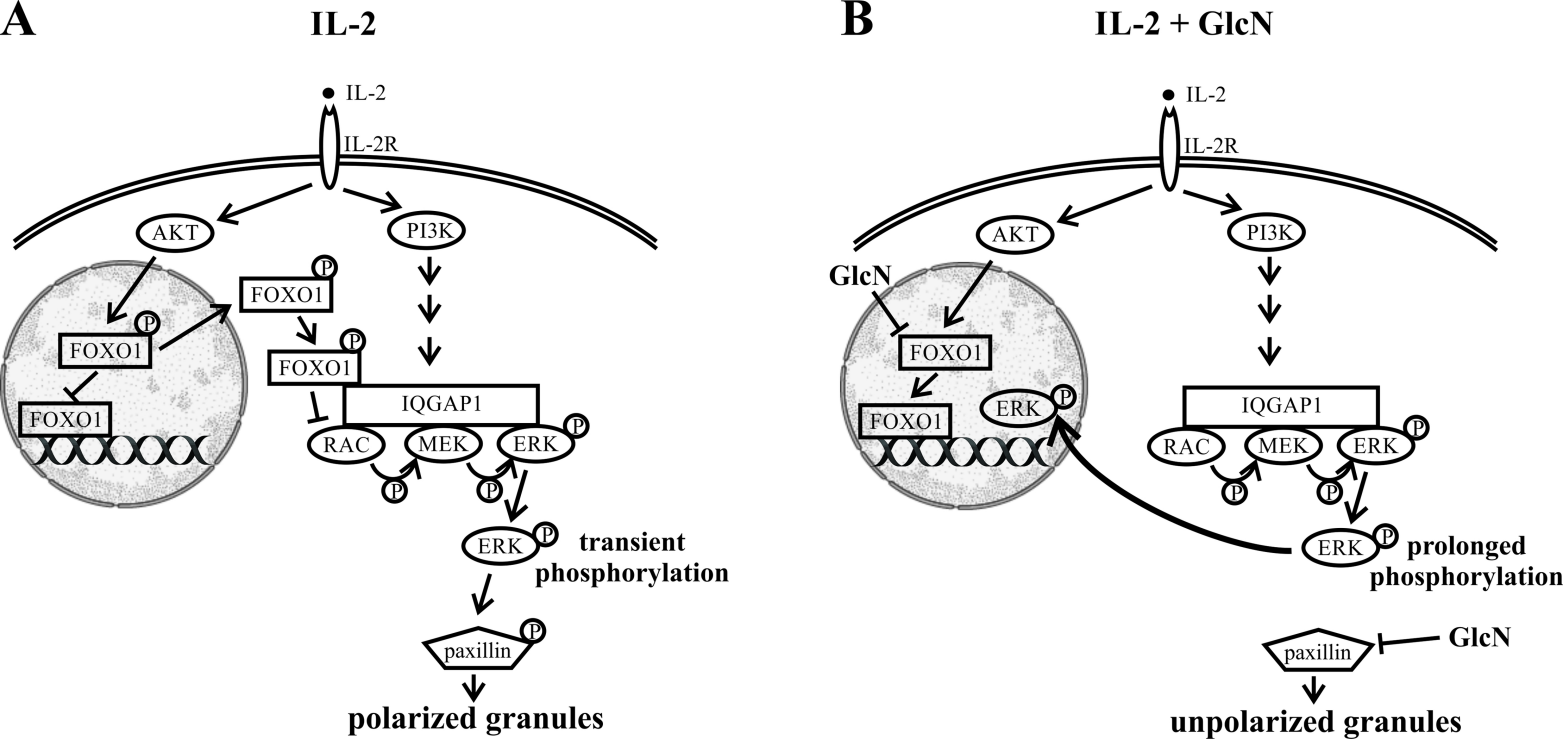
Schematic Illustration of Glucosamine (GlcN) Effects on Granule Polarization in NK-92 Cells. (A) A simplified scheme of the IL2-activated granule polarization. IL2 treatment of NK-92 cells induces a strong activation of AKT kinase and the PI3K pathway. PI3K indirectly phosphorylates ERK by forming a complex with IQGAP1, RAC, MEK, and ERK. Simultaneously, AKT kinase triggers phosphorylation of FOXO1, which is then translocated to the cytosol. In the cytosol, FOXO1 suppresses ERK phosphorylation by binding to IQGAP1, leading to transient phosphorylation of ERK. Paxillin is phosphorylated by ERK, and this phosphorylation regulates granule migration. (B) GlcN inhibits FOXO1 phosphorylation, resulting in its nuclear localization, which further promotes prolonged phosphorylation of ERK, resulting in translocation of ERK into the nucleus. Additionally, GlcN prevents phosphorylation of paxillin, leading to appearance of unpolarized granules and immunosuppression of NK-92 cells.

Moreover, GlcN affects the localization of granules, and our results showed that GlcN interferes with the movements of granules toward the immunological synapses, thus affecting the cytotoxicity of NK-92 cells. Granule polarization, driven by MTOC reorientation, plays an important role in the activity of NK cells [45, 55]. This process includes the signaling events associated with members of the MAPK family [44–46]. A simplified schematic representation of the granule polarization mechanism is presented in Fig 7A, based on the information on the molecular organization [19, 55–57] in similar cell systems and our results obtained using the IL2-activated NK-92 cells. Activation of JNK, ERK, and P38 in NK cells varies depending on the stimulated receptor. We showed that IL2 treatment leads to rapid phosphorylation of ERK, but neither P38 kinase nor JNK was phosphorylated under these conditions, and therefore, we were able to monitor the effects of GlcN on the AKT-PI3K-ERK pathway. High concentrations of IL2 were shown to induce transient activation of ERK in the NK cells, accompanied by their cytotoxic activities. Although the mechanisms underlying the effects of NK-92 cells on target cells remain to be completely elucidated, GlcN treatment was shown to induce a prolonged phosphorylation of ERK in the IL2-stimulated NK cells through modification of the serine residue phosphorylation in the transcription factor FOXO1. Prolonged ERK phosphorylation leads to translocation of this protein to the nucleus (Fig 5), where it targets transcription factors that regulate expression of the immediate early genes and proteins and chromatin remodeling, among other processes [58]. This may help explain why the granules are not focused on the immune synapse in presence of GlcN. GlcN-associated inhibition of polarization occurs downstream of ERK or JNK, at the level of paxillin [17, 19, 59]. We showed that increase in ERK nuclear localization and extensive differential *O*-GlcNAc modification are associated with inhibition of paxillin phosphorylation, which results in altered migration of granules and a decrease in NK cell activity. In Fig 7B, we present the mechanisms underlying the immunosuppressive effect of GlcN on NK-92 cells.

In conclusion, we demonstrated that GlcN-associated inhibition of NK-92 cell cytotoxicity is based on alterations in granule localization and prevention of their polarization in the direction of immunological synapse, and not on the inhibition of the activity of cathepsins C, L, and E. GlcN modulates the activity of kinases responsible for NK cell functions against cancer cells. To further clarify and confirm the proposed mechanism of GlcN action on NK cell cytotoxicity, additional experiments *in vivo* have to be performed in the future.
